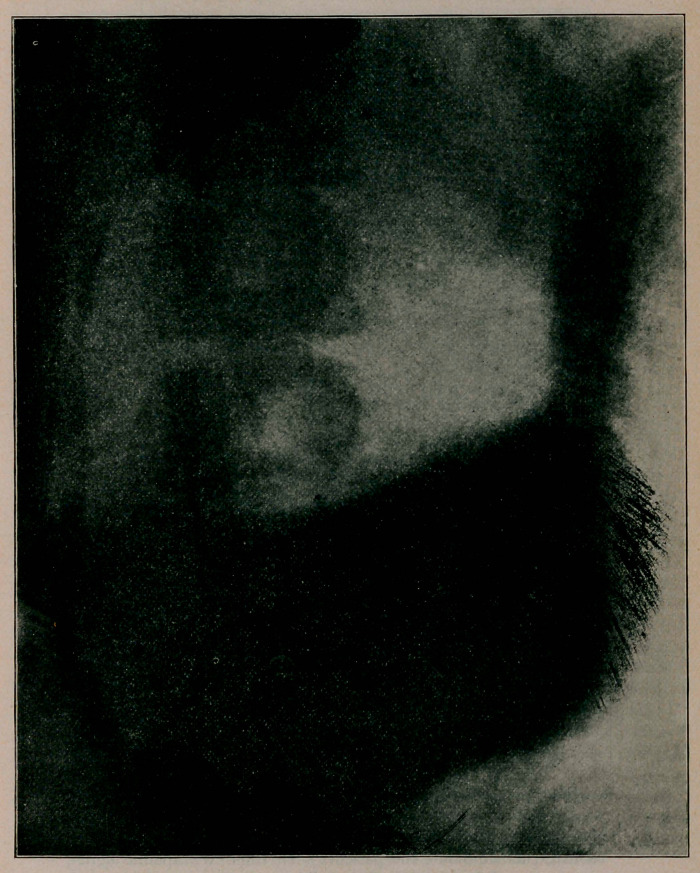# Syphilis of the Stomach

**Published:** 1912-12

**Authors:** 


### ABSTRACTS.

Syphilis of the Stomach, Jerome Meyers, *Albany Med. Annals,* Oct., 1912. Male, 24, skin lesions and Wassermann reaction positive. Muscular swelling in epigastrium. Stomach 2 fingers' breaths below umbilicus recumbent, 5 standing. Radiograph shows dilated and sagging stomach. Improvement under KI. The author gives an extensive review of literature and tabulation of cases to date. Articles of this kind are of the highest value, being complete monographs which should be saved for reference. Through the courtesy of the editor, we reproduce Dr. Meyer's radiogram.

## Figures and Tables

**Figure f1:**